# Genome-Wide Massively Parallel Sequencing of Formaldehyde Fixed-Paraffin Embedded (FFPE) Tumor Tissues for Copy-Number- and Mutation-Analysis

**DOI:** 10.1371/journal.pone.0005548

**Published:** 2009-05-14

**Authors:** Michal R. Schweiger, Martin Kerick, Bernd Timmermann, Marcus W. Albrecht, Tatjana Borodina, Dmitri Parkhomchuk, Kurt Zatloukal, Hans Lehrach

**Affiliations:** 1 Department of Vertebrate Genomics, Max Planck Institute for Molecular Genetics, Berlin, Germany; 2 Institute for Medical Genetics, Charité Universitätsmedizin, Berlin, Germany; 3 Institute of Pathology, Medical University, Graz, Austria; Georgia Institute of Technology, United States of America

## Abstract

**Background:**

Cancer re-sequencing programs rely on DNA isolated from fresh snap frozen tissues, the preparation of which is combined with additional preservation efforts. Tissue samples at pathology departments are routinely stored as formalin-fixed and paraffin-embedded (FFPE) samples and their use would open up access to a variety of clinical trials. However, FFPE preparation is incompatible with many down-stream molecular biology techniques such as PCR based amplification methods and gene expression studies.

**Methodology/Principal Findings:**

Here we investigated the sample quality requirements of FFPE tissues for massively parallel short-read sequencing approaches. We evaluated key variables of pre-fixation, fixation related and post-fixation processes that occur in routine medical service (e.g. degree of autolysis, duration of fixation and of storage). We also investigated the influence of tissue storage time on sequencing quality by using material that was up to 18 years old. Finally, we analyzed normal and tumor breast tissues using the Sequencing by Synthesis technique (Illumina Genome Analyzer, Solexa) to simultaneously localize genome-wide copy number alterations and to detect genomic variations such as substitutions and point-deletions and/or insertions in FFPE tissue samples.

**Conclusions/Significance:**

The application of second generation sequencing techniques on small amounts of FFPE material opens up the possibility to analyze tissue samples which have been collected during routine clinical work as well as in the context of clinical trials. This is in particular important since FFPE samples are amply available from surgical tumor resections and histopathological diagnosis, and comprise tissue from precursor lesions, primary tumors, lymphogenic and/or hematogenic metastases. Large-scale studies using this tissue material will result in a better prediction of the prognosis of cancer patients and the early identification of patients which will respond to therapy.

## Introduction

The advance of second generation sequencing techniques (454 sequencing, the Illumina Genome Analyzer (Solexa), the SOLiD platform, the Polonator and the HeliScope Single Molecule Sequencer technology) enables us to investigate many “-omes” of patients material, such as genomes, transcriptomes, epigenomes and miRNAomes [1], [2], [3]. The sequencing techniques are based on the amplification of single DNA molecules and thereby provide, in a sense, ‘digital’ information [4], [5], [6]. They are considerably less error-prone to the ‘contamination’ between normal and diseased tissues and are especially advantageous for the analysis of tumor tissues [7].

To date, cancer sequencing programs have relied on DNA isolated from fresh frozen tissues, access to which can be limited [8]. The use of preserved material, i.e. from tissue banks, could help to avoid this limitation and would enable the reanalysis of diverse clinical trials. A common and convenient way to store tissues over long periods of time is to fix them in formaldehyde and embed them in paraffin. Large collections of diseased and normal tissues are stored at hospitals and provide an excellent source for molecular genetic studies. However, the recovery of nucleic acids from these tissue specimens is challenging. Not only does formaldehyde lead to crosslinkages between DNA or RNA and proteins, but it also results in the preparation of fragmented DNA. Both factors restrict diverse genetic techniques like polymerase chain reactions due to poor quantity, purity and length of the RNA and DNA.

To take advantage of preserved patient tissue we investigated the sample quality requirements of formaldehyde-fixed and paraffin-embedded (FFPE) tissues and established a protocol for the application of massively parallel short-read sequencing approaches. We evaluated key variables of pre-fixation, fixation related and post-fixation processes that occur in routine medical service (e.g. degree of autolysis, duration of fixation and of storage) (Table 1). We also investigated the influence of tissue storage time on sequencing quality by using material that was up to 18 years old. Finally, we analyzed normal and tumor breast tissues using the Sequencing by Synthesis (Illumina Genome Analyzer) technology to simultaneously localize genome-wide copy number alterations and to detect genomic variations such as substitutions and point-deletions and/or insertions in FFPE tissue samples.

**Table 1:** Snap frozen and FFPE tissue samples used for sequencing.

Sample ID	Tissue	Age (years)	Ischemia (min.)	Fixation method
ID-1.0	Breast - normal	1	<20	Snap frozen
ID-1.1	Breast - normal	1	<20	24 hrs formaldehyde
ID-1.3	Breast - normal	1	60	24 hrs formaldehyde
ID-1.7	Breast - normal	1	360	24 hrs formaldehyde
ID-1.6	Breast - normal	1	180	72 hrs formaldehyde
ID-1.8	Breast - normal	1	360	72 hrs formaldehyde
ID-22.1	Breast - normal	18	<20	Snap frozen
ID-22.2	Breast - normal	18	<20	24 hrs formaldehyde
ID-02.15	Breast -tumor	14	<20	Snap frozen
ID-02.48	Breast - tumor	14	<20	24 hrs formaldehyde

## Results

### Genome-wide parallel sequencing of snap frozen and FFPE tissue DNA

**Figure 1:**
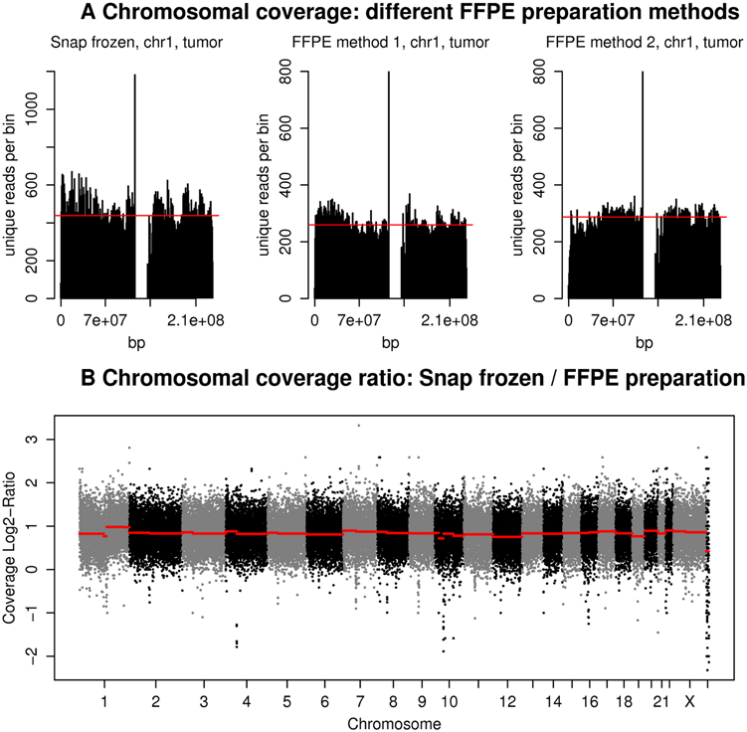
Distribution of sequencing fragments for DNA extracted from snap frozen and FFPE breast tissues. (A) Shown are the distributions of sequencing fragments over chromosome 1 using three different tissue conservation techniques Left: Snap frozen tissues, middle: FFPE tissue with 20 min. ischemia, and right: FFPE tissue with 60 min. ischemia. The red line depicts median genome coverage. (B) Chromosomal coverage ratio of snap frozen versus FFPE preparations of normal tissue samples. Each chromosome has been split into equal sized bins of 50 Kb size. The log2 ratios of unique reads per bin have been plotted across all chromosomes.

For the comparison of snap frozen and FFPE tissue DNA we prepared breast tissue samples from one patient, divided the tissue in seven equal amounts and preserved them either as snap frozen or as FFPE tissue with ischemic times ranging from 20 min. to over 6 hours and fixation times from 24 to 72 hours (Table 1). After six months storage time, DNA was extracted, quality controlled by aid of a Bioanalyzer array and sequenced with the Sequencing by Synthesis technology (SBS). Test DNA extractions using the QIAamp, Arcturus PicoPure and Macherey-Nagel DNA extraction kits yielded equally successful sequencing results and therefore further experiments were performed according to the Qiagen protocol (data not shown). Tissue storage conditions followed those used most frequently in routine pathology department protocols. The sequencing resulted in approximately 3 to 5 million reads with 2 to 3 million mappable unique fragments per sample (Table 2). For a visualization of the fragment distributions we divided each chromosome in 300 equal sized parts (‘bins’) and calculated the fragment number for each bin. Using these 300 bins per chromosome we found a very similar distribution of fragments over all chromosomes (Figure 1) for snap frozen as well as for FFPE tissue DNA. Comparing the readcounts per set unit (bin) for snap frozen and FFPE tissue as a measure of coverage coherence, we calculated the Pearson correlation coefficient and found a median correlation of bincounts of 0.82 for snap frozen and FFPE tissues. We also used genomic bin sizes of 50, 100, 250, 500 and 1000 Kb and stratified the correlation coefficients by the GC content of the bins (Supplementary Figure S1). Bins with the most common GC content of approximately 40% have correlation values of approximately 0.97. The dependency of correlation values from GC content is decreasing with larger bin sizes though and is therefore likely a consequence of low coverage. Similar findings were observed for technical replicates (Supplementary Figure S2). In both cases the majority of correlation coefficients are above 0.6 and the variability decreases with increasing bin sizes. Thus, overall fragment numbers, aligned fragments and chromosome-wise coverage are similar for snap frozen and FFPE tissues, proving that fresh FFPE tissues can be used for high throughput re-sequencing approaches.

**Table 2:** Sequencing results, NE in snap frozen and FFPE tissues.

Sample ID	Reads (million)	unique aligned reads (million)	SNPs	known SNPs	common SNPs (%)
ID-1.0	3,217	2,399	3419	1128	
ID-1.1	3,217	1,373	6804	1461	81.4
ID-1.3	2,604	1,621	5413	1261	83.9
ID-22.1	3,296	2,369	9503	1567	
ID-22.2	3,047	1,509	12734	1495	87.3
ID-02.15	2,074	1,521	2839	955	
ID-02.48	2,631	0.92	6348	1303	89.8

For each set, ‘common SNPs’ were calculated in relation to the snap frozen samples.

### Sequencing of up to 18 years old preserved tissue material

Most of the tissues stored in biobanks are several years old and clinical trials date back to at least ten year collection times. Therefore, it would be highly desirable to include tissue samples that have been stored long-term for genome-wide sequencing techniques. In addition, studies of rare clinical diseases such as Von Hippel-Lindau or Li-Fraumeni syndrome would become more feasible to perform. Sequencing by Synthesis (SBS) of snap frozen - and FFPE preserved tissue samples that were 14 and 18 years old resulted in approximately 1 million and 4 million aligned reads with an even distribution over all chromosomes. Median correlation of bincounts for snap frozen and FFPE tissue was found to be very similar to recent tissue values at 0.84. Thus, tissue storage time has a minor influence on sequence quality. It would be interesting to analyze even older material in the same manner.

### Detection of CNA and nucleotide-specific genomic alterations in breast tumors

**Figure 2:**
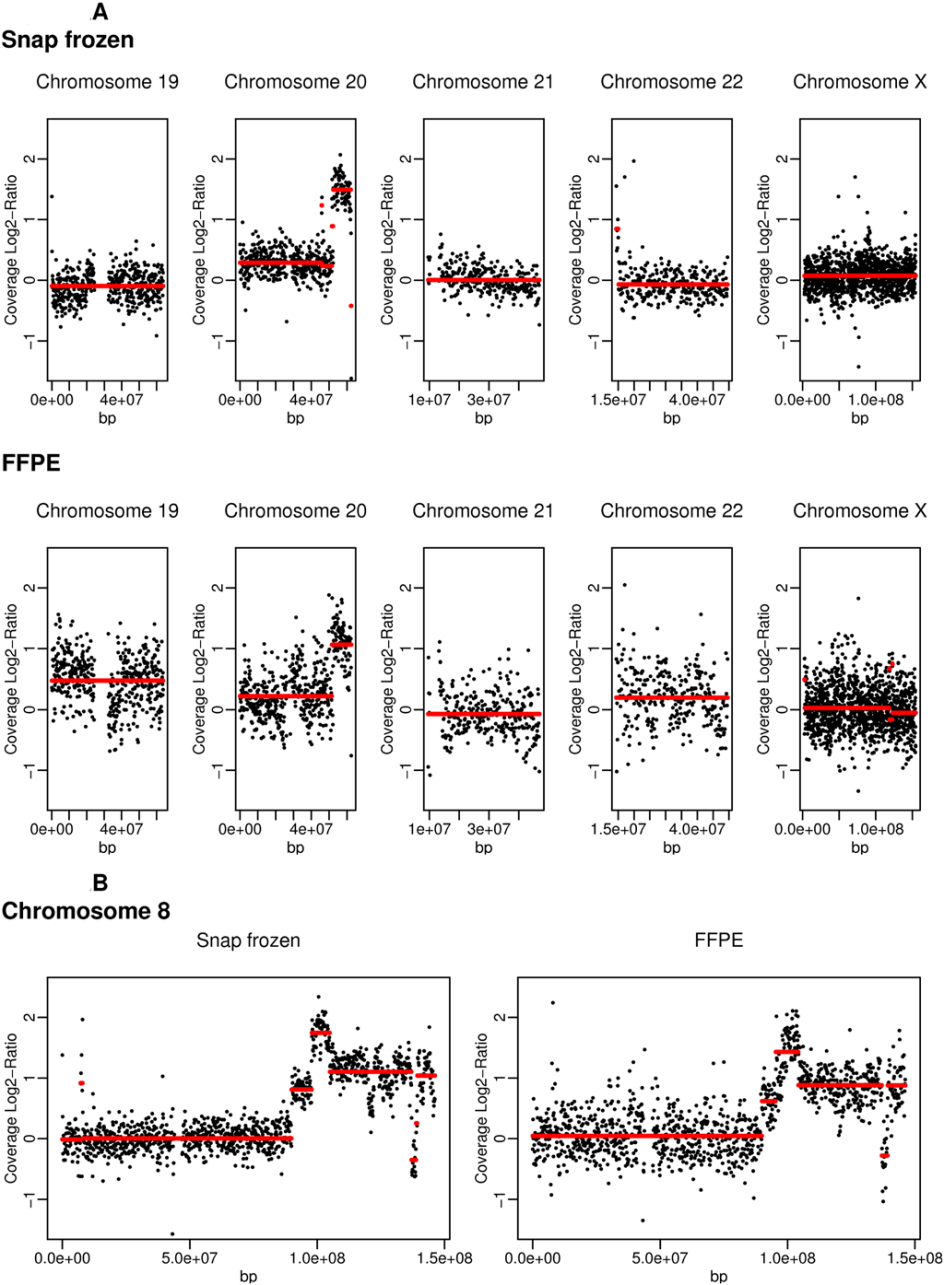
Copy number variations in DNA from snap frozen and FFPE cancer tissues in relation to normal tissue. (A) DNA fragment coverage ratio of tumor versus normal tissue on five chromosomes. The x-axis represents the genomic position. The y-axis represents the log2 ratio of fragments per bin of tumor versus normal tissue. The fragment numbers are calculated per 120 Kb segment. The red lines depict the local averages as calculated by DNAcopy [12]. Local differences in copy numbers exceeding two standard deviations are highlighted. (B) Detailed view of the fragment distribution of chromosome 8 from snap frozen and FFPE breast cancer tissue samples.

In addition to normal tissue samples, we also used tumor tissue samples to investigate copy number alterations (CNA) and mutation frequencies. CNAs are useful parameters for tumor classifications and – as recently introduced in genetic counseling procedures – as diagnostic tool for children with mental retardation of unknown origin [9], [10]. The identification of tumor mutations is essential for the layout of a calculated chemotherapy. For example, tyrosine kinase inhibitors (TKIs), such as gefitinib and erlotinib, are only efficient in patients without K-ras mutations [11]. Up to now the detection of CNAs and mutations required that two different techniques be employed in routine clinical diagnostics: CNAs are determined by comparative genomic hybridization (CGH) arrays and mutations are found by conventional sequencing techniques. With the power of second generation sequencing techniques, both sets of information will be achieved in one process. Along this line, we provide here the combined data for breast tumor tissues, both snap frozen as well as FFPE tissues, using single SBS approaches. For these experiments, breast tumor tissues were enriched for at least 70% tumor cell content by macro-dissections. DNA libraries for the Illumina Genome Analyzer (Solexa) were then prepared and sequenced. We received approximately 1 million reads with 0.5 million unique mappable fragments. Using the DNAcopy software in R [12] we found gains of DNA on chromosome 8 and 20 (Figure 2A). For chromosome 8, a fine-mapping of copy number alterations revealed gain of DNA between the chromosomal localizations 90.32 Mb and 146.27 Mb with a small disruption between 137.06 Mb–139.4 Mb (Figure 2B). The CNAs found were identical for snap frozen - and FFPE – material. They also corresponded to frequent alterations found in breast tumors in general, underlining the reliability of the sequencing technique [13].

Using one eighth of each Illumina Genome Analyzer (Solexa) run sequencing capacity, the resolution with regard to CNAs lies at approximately 15 Kb with 10 fragments per bin and can be easily improved by additional sequencing. In comparison, the resolution of the 244 K Array from Agilent, a frequently used array in clinical settings, lies at approximately 45 Kb with 5 oligos. In addition to CNA information, second generation sequencing allows one to investigate genomic alterations, such as substitutions, deletions and insertions and therefore will be a basic tool for routine clinical diagnostics in the future.

**Figure 3:**
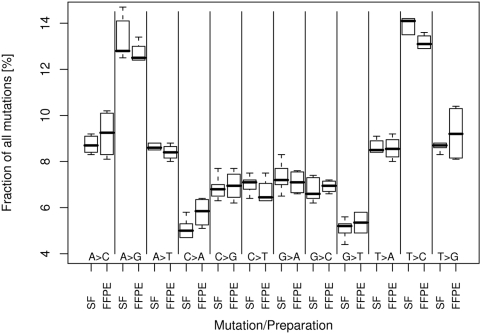
Nucleotide exchange rates in DNA from snap frozen and FFPE tissues. Using the Maq 0.7.1 software nucleotide exchanges were calculated for all possible transitions (e.g. A>G, A>C) for four snap frozen (SF) and five FFPE tissue samples [18]. Shown are boxblots with NE rates as fractions of all mutations found per patient sample.

In addition to CNAs, we also calculated nucleotide exchange (NE) rates for all samples. In general, we found a NE probability of 0.1 to 0.5% per aligned sequence read (Table 2). Approximately 12% to 33% of NEs found were located in dbSNP and can therefore be classified as known SNPs [14]. We also compared the NEs pairwise under consideration of an at least 8fold coverage and identified up to 90% ‘common SNPs’ for samples ID-02.15 and ID-02.48 (Table 2). The absolute amount of called SNPs appears to be higher for FFPE tissues with a lower rate of known SNPs identified. This is most likely due to DNA damage of the FFPE samples. By increasing the sequence depth of the samples the overlap between NEs for snap frozen and FFPE DNA increases, suggesting that with an increase of sequence coverage the rate of false positive calls in FFPE can be minimized (Supplementary Figure S3). The amount of specific NE rates such as A to G or C to A varies between 5% and 14%. However, the amounts of specific NE rates were comparable in different sequencing settings with an average standard deviation of 0.7%. The reproducibility of specific NE rates was also found in the comparison of snap frozen and FFPE tissue DNA as shown in Figure 3 and Table 3. A higher variability of NEs in FFPE tissue samples is largely due to extended fixation times since we have included all available sequencing data from table 1 in our calculations. For both tissue preparation techniques we found corresponding NE rates, suggesting that FFPE tissues can also be used for mutational analyses. Filtering for homozygous mutations, we found approximately 5% of all SNPs with sequencing coverage rates greater than 8-fold and we were able to repeatedly detect approximately 90% of the mutations in both snap frozen and FFPE tissue preparations. As expected, the absolute amount of known SNPs increased with the number of sequencing fragments aligned for both snap frozen as well as FFPE tissues. This again demonstrates the applicability and scalability of second generation sequencing techniques for the detection of mutations in FFPE tissues.

**Table 3:** NE for samples ID-1.0 (snap forzen) and ID-1.1 (FFPE).

NE	Snap Frozen		FFPE	
	Count	Fraction	Count	Fraction
A→C	290	8.4%	582	8.5%
A→G	506	14.7%	866	12.6%
A→T	257	7.5%	570	8.3%
C→A	173	5.0%	429	6.3%
C→G	235	6.8%	529	7.7%
C→T	243	7.1%	435	6.3%
G→A	266	7.7%	512	7.5%
G→C	226	6.6%	495	7.2%
G→T	150	4.4%	396	5.8%
T→A	232	6.7%	547	8.0%
T→C	571	16.6%	935	13.6%
T→G	296	8.6%	566	8.2%
	3445		6862	

Taken together, our results illustrate that DNA extracted from FFPE tissue can be used for second generation re-sequencing approaches. We also demonstrated the possibility of gathering information about CNAs and mutations with one single sequencing effort from snap frozen as well as FFPE preserved tissue types and proved that the data obtained from both preparation methods is of comparable quality.

## Discussion

In this paper, we investigated if new and archived FFPE tissues could be used in addition to snap frozen tissues for massively parallel sequencing and what requirements need to be fulfilled for their use. We conclude that DNA extracted from FFPE tissues can be used for Sequencing by Synthesis (SBS) and that neither an increase in ischemia time from 20 min. to over 360 min. nor a longer fixation time of up to 72 hours plays a major role in sequencing quality (Figure 1, Table 1). Overall, the percentage of unique mappable reads was in the same range for snap frozen and paraffin-embedded samples. In general, we see variations in mappable reads independent of the DNA source. However, for FFPE samples the variability might also be partly due to DNA damages resulting in more fragments with one or more mismatches. Even though the absolute numbers of unique alignments is slightly smaller for DNA extracted from FFPE tissues, the fragments are evenly distributed over the genome and cover all chromosomes. Furthermore, as demonstrated, FFPE tissue samples stored over 18 years in pathology departments can be successfully sequenced and integrated in re-sequencing programs. This provides an excellent opportunity to evaluate tissue samples which have been collected and stored in biobanks for clinical trials with anti cancer drugs. For instance, it has become evident that the response to inhibitors of receptor tyrosine kinases or inhibitors of downstream signaling molecules not only depends on the expression of the drug target itself but also on the presence of mutations or polymorphisms in functional domains of the target [15] and its down stream signaling network [11].

The power of large-scale sequencing techniques not only lies in the large amount of sequencing throughput but also in the possibility to gain multi-faceted data of genomic, epigenomic and transcriptomic origin.

As an example of the type of genome-based information that can be obtained, we presented the successful calculation of copy number alterations and mutations from snap frozen as well as from FFPE breast tumor tissue. Copy number alterations in breast cancer tissue were found equally well in snap frozen and FFPE prepared tissue (Figure 2).

A patient-wise comparison of snap frozen and FFPE tissues with regard to called mutations demonstrated concordance for 81 to 95% of mutations found in both preparation types given an eightfold coverage rate. The comparable mutation rates and frequencies for each transition and transversion again demonstrated the reproducibility of the sequencing results in different preparation settings (Table 2). The detection limit depended upon the coverage rate calculated for technical replicates of snap frozen and FFPE tissues (data not shown), indicating that the number of consistently detected mutations in both tissue preparations could be increased beyond 90%.

Formalin-fixed and paraffin-embedded tissue has not been used in large-scale sequencing protocols. Our study demonstrated that it can be reliably employed for highly relevant clinical problems such as the combined investigation of CNAs and SNPs in scarce tissue samples.

Our observation that long-term storage of samples has no significant negative effect on sequencing results implies that it is possible to re-evaluate FFPE-samples from participants in previous clinical trials. In this way, DNA sequence-based biomarkers might be established which discriminate responders and non-responders to therapy, thereby contributing to a better targeted and more efficacious application of new therapeutic treatments. In addition, the determination of CNAs as well as mutations is not only essential for tumor diagnosis, but is also required for the diagnosis of a diverse array of genetic disorders associated with deletions or gains of DNA such as trisomy 21 and microdeletions-syndrome CATCH22 or with mutations such as Marfan's Syndrome.

In summary, our results illustrate that DNA extracted from FFPE tissue can be used for copy number analysis as well as mutation analysis. Determining CNAs and mutations with one single sequencing effort renders our method very efficient in terms of time and money. The applicability and scalability of second generation sequencing techniques is a major improvement for the analysis of archived tissue material from biobanks and will lead to the application of large-scale sequencing techniques as routine diagnostic tools in general genetic counseling and oncology.

## Materials and Methods

### Ethics statement

The study has been approved by the Ethical Committee of the Medical University of Graz (ethics statement #20-066 signed by Prof.Dr. P.H. Rehak and Prof. DDr. H.P. Kapfhammer). For new samples patients have given their written informed consent. For old samples (14 and 18 years old) no informed consent was available, therefore all samples and medical data used in this study have been irreversibly anonymized.

### Tissue storage

Human tissues obtained during surgery were divided into several aliquots which were either snap frozen in methyl butane at the temperature of liquid nitrogen or fixed in 4% buffered (pH 7.4) formaldehyde for 24 and 72 hrs and embedded in paraffin using an automated embedding system immediately after surgery (<20 min cold ischemia time). To simulate the impact of cold ischemia – samples were kept for 1 hr, 3 hrs and 6 hrs in a humidified chamber at room temperature before freezing. The impact of long-term storage (1 yr compared to 14 yrs and 18 yrs) of FFPE samples was investigated in samples retrieved from the tissue bank of the Institute of Pathology [16].

### DNA preparation

From frozen tissue samples cryo sections of 3 µm thickness were produced and stained with haematoxylin eosin to evaluate tumor cell content. Further cryo sections were collected and processed for DNA isolation using the QIAamp DNA Mini Kit (Qiagen), according to the manufacturer's instructions.

DNA was isolated from FFPE samples after extraction of 3 µm thick paraffin sections in xylene and by using the QIAamp DNA Mini Kit (Qiagen) PicoPure DNA Extraction Kit (Molecular Devices) and the Macherey-Nagel DNA extraction protocol following the same protocol as applied for frozen samples.

### Illumina Genome Analyzer (Solexa) library preparation and sequencing

Preparation of the libraries and sequencing were performed using the Solexa sequencing platform (GenomeAnalyzer, Illumina) following the manufacturer's instructions. We randomly sheared 1,5 µg genomic DNA to fragments less than 800 bp. The fragmented DNA was end-repaired using T4 DNA polymerase and Klenow polymerase with T4 polynucleotide kinase activity to blunt end and phosporylate the 5′ ends. A 3′ overhang was created using a exonuclease negative Klenow fragment and Illumina adaptor oligonucleotides were ligated to the sticky ends. Ligation products were purified by an agarose gel electrophoresis and enriched for successfully ligated fragments with an 18-cycle PCR using sequencing primers on either end. Ligation products were then used for cluster generation and sequencing- by- synthesis using the Illumina Genome Analyzer (Solexa) .

### Bioinformatics data analysis

Image Analysis and base calling were performed using Firecrest 1.9.5_14 and Bustard 1.9.5_14 and reads were aligned to the human genome (NCBI36) using Bowtie 0.9.7.1 [17]. The chromosomal coverage was determined by splitting each chromosome in equally sized regions (bins) and counting the reads that mapped equally to each bin. The coverage comparison of different FFPE preparation protocols was visualized using 300 bins per chromosome.

To compare the read coverage of different samples across all chromosomes we counted uniquely mapped reads for five different binsizes (50 Kb, 100 Kb, 250 Kb, 500 Kb, 1 Mb). As metric of comparison we used the Pearson correlation coefficient on the counts per bin. In detail, we determined the Pearson coefficient for each chromosome separately and calculated the median across all chromosomes. Stratification of bincount correlation by GC content was done by calculating the Pearson correlation coefficient across all bins of a certain GC content independent of chromosomal location.

Copy number analysis was done in R using the DNAcopy package [12]. In short, DNA read frequencies were determined for bins of 120 Kb. The log2 frequency ratio of corresponding bins was calculated for tumor versus normal tissue. Median of ratios was centered to zero experiment wise. Log ratios were smoothed by DNAcopy using default values and copy number variation was detected by DNAcopy using a threshold of two standard deviations.

Overall nucleotide exchanges were calculated using the Maq 0.7.1 software with default parameters for the steps cns2snp & SNPfilter [18]. Homozygous mutations detected in snap frozen and FFPE prepared material with at least 8-fold coverage in both samples were then compared patient wise. For the determination of the detection limit homozygous mutations in technical replicates for snap frozen and FFPE were compared.

## Supporting Information

Figure S1 Dependency of coverage correlation coefficients of snap frozen and FFPE tissues on GC content and bin size. The GC content and the corresponding coverage correlation coefficients for bin sizes of 50, 100, 250, 500 and 1000 Kb were calculated for snap frozen and FFPE tissues. Shown is one plot for each bin size with the GC content on the x-axis, the Pearson correlation coefficients as circles and the GC content per bin-distribution as a histogram. A locally weighted polynomial regression (LOESS) has been fitted to visualize trends.
(0.51 MB TIF)

Figure S2 Dependency of coverage correlation coefficients from two technical replicates on GC content and bin size. The GC content and the corresponding coverage correlation coefficients for bin sizes of 50, 100, 250, 500 and 1000 Kb were calculated for two technical replicates. Shown is one blot for each bin size with the GC content on the x-axis, the Pearson correlation coefficients as circles and the GC content per bin-distribution as a histogram. A locally weighted polynomial regression (LOESS) has been fitted to visualize trends.
(0.50 MB TIF)

Figure S3 The amount of SNPs found common in FFPE and snap frozen preparations increases with higher coverage rates. Intersections between two samples were calculated under consideration of 4-fold, 8-fold and 12-fold coverages. Open triangle: ID1.0 against ID1.3, closed circles: ID1.0 vs ID1.1, closed rectangle: ID22.1 against ID 22.2, Open rectangle: ID-02.15 against ID-02.48, stars: ID-02.14 against ID-02.58.
(0.11 MB TIF)
