# Supplementary figures and images for: Genome-Wide Massively Parallel Sequencing of Formaldehyde Fixed-Paraffin Embedded (FFPE) Tumor Tissues for Copy-Number- and Mutation-Analysis
Source: PLoS One. 2009 May 14;4(5):e5548. doi: 10.1371/journal.pone.0005548 (PMC2678265; doi:10.1371/journal.pone.0005548)

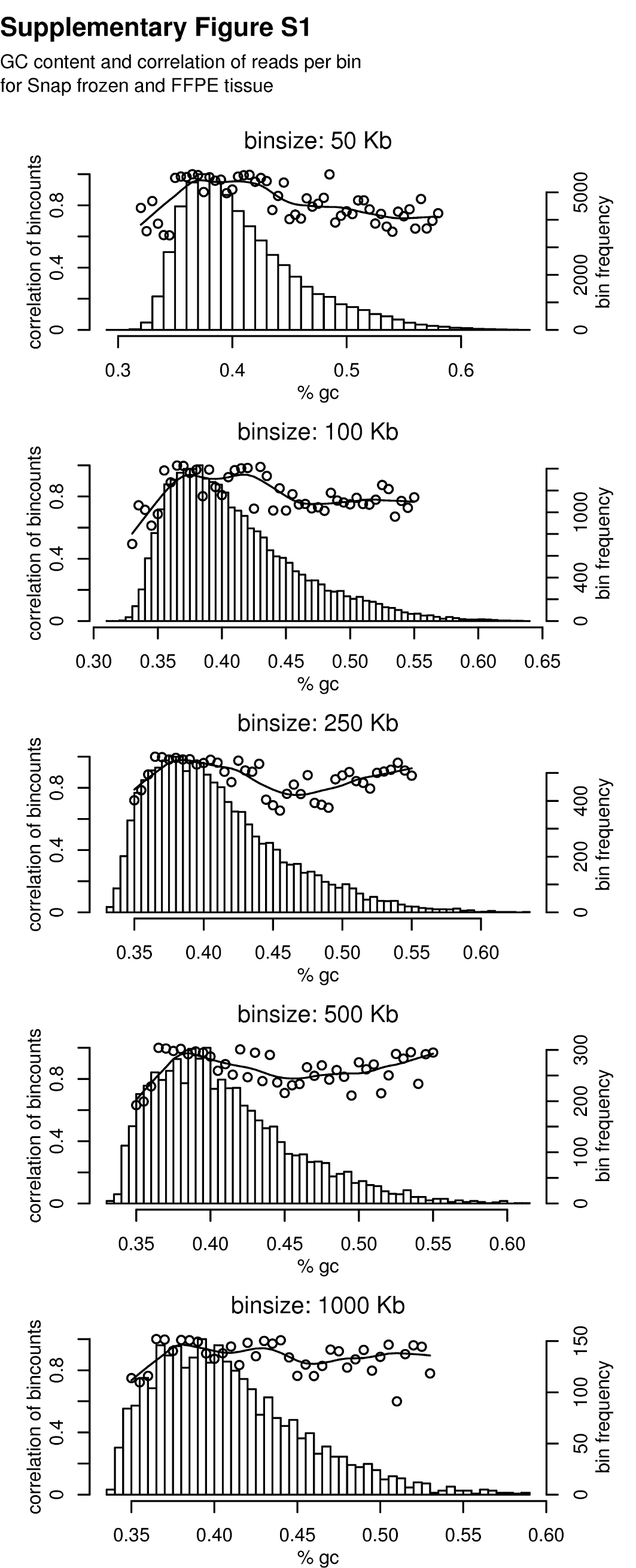

Supplement: Figure S1 — Dependency of coverage correlation coefficients of snap frozen and FFPE tissues on GC content and bin size. The GC content and the corresponding coverage correlation coefficients for bin sizes of 50, 100, 250, 500 and 1000 Kb were calculated for snap frozen and FFPE tissues. Shown is one plot for each bin size with the GC content on the x-axis, the Pearson correlation coefficients as circles and the GC content per bin-distribution as a histogram. A locally weighted polynomial regression (LOESS) has been fitted to visualize trends. (0.51 MB TIF) [file pone.0005548.s001.tif]

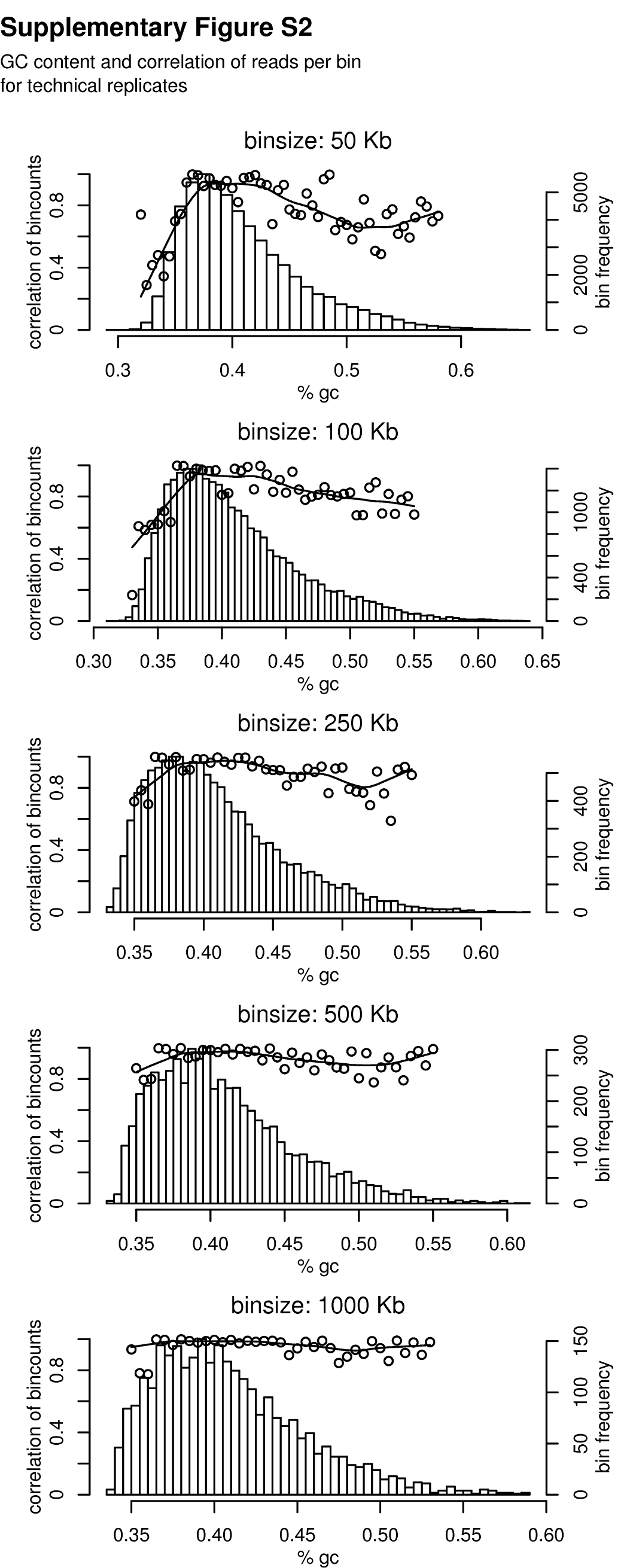

Supplement: Figure S2 — Dependency of coverage correlation coefficients from two technical replicates on GC content and bin size. The GC content and the corresponding coverage correlation coefficients for bin sizes of 50, 100, 250, 500 and 1000 Kb were calculated for two technical replicates. Shown is one blot for each bin size with the GC content on the x-axis, the Pearson correlation coefficients as circles and the GC content per bin-distribution as a histogram. A locally weighted polynomial regression (LOESS) has been fitted to visualize trends. (0.50 MB TIF) [file pone.0005548.s002.tif]

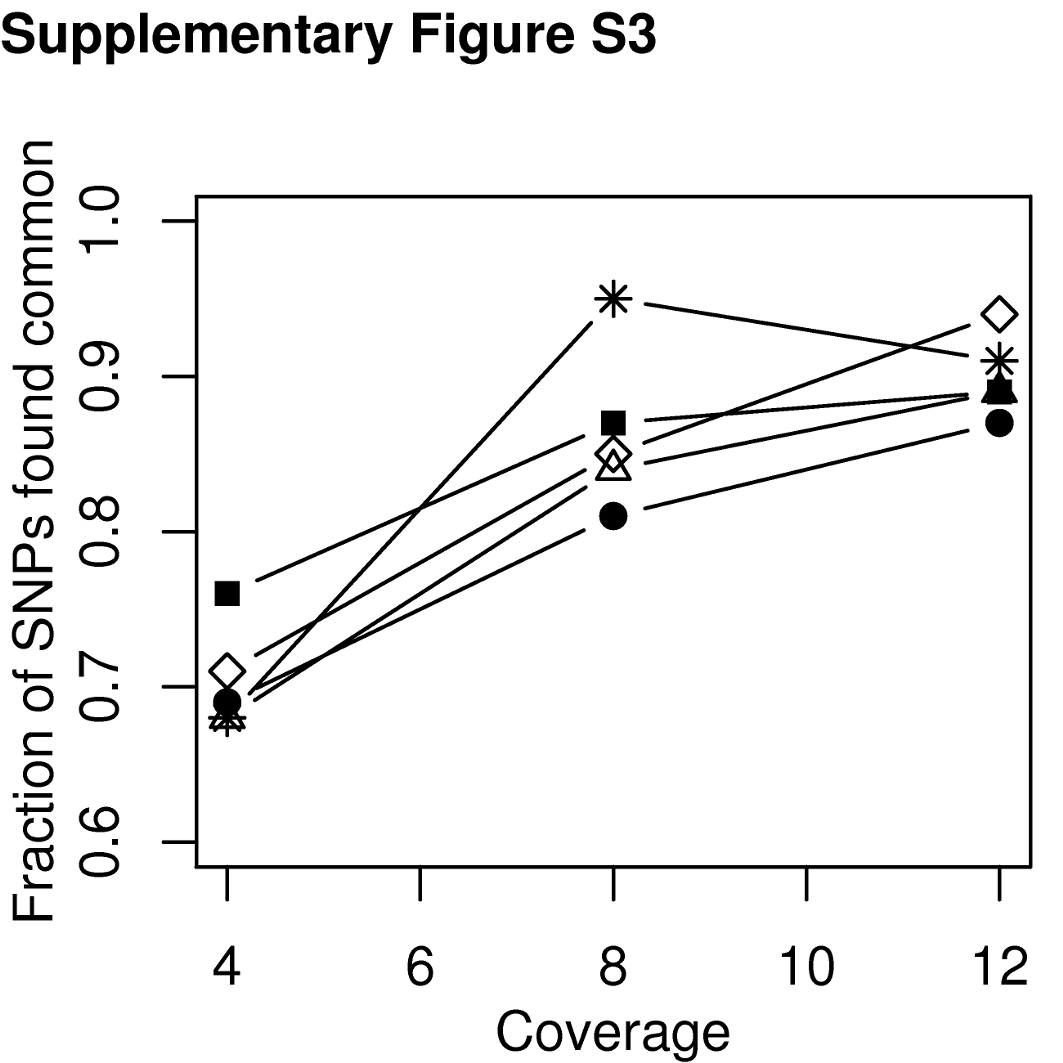

Supplement: Figure S3 — The amount of SNPs found common in FFPE and snap frozen preparations increases with higher coverage rates. Intersections between two samples were calculated under consideration of 4-fold, 8-fold and 12-fold coverages. Open triangle: ID1.0 against ID1.3, closed circles: ID1.0 vs ID1.1, closed rectangle: ID22.1 against ID 22.2, Open rectangle: ID-02.15 against ID-02.48, stars: ID-02.14 against ID-02.58. (0.11 MB TIF) [file pone.0005548.s003.tif]
